# Adsorption/Coagulation/Ceramic Microfiltration for Treating Challenging Waters for Drinking Water Production

**DOI:** 10.3390/membranes11020091

**Published:** 2021-01-27

**Authors:** Margarida Campinas, Rui M. C. Viegas, Rosário Coelho, Helena Lucas, Maria João Rosa

**Affiliations:** 1 Water Quality and Treatment Laboratory, Urban Water Unit, Hydraulics and Environment Department, LNEC—National Civil Engineering Laboratory, 1700-066 Lisbon, Portugal; rviegas@lnec.pt (R.M.C.V.); mjrosa@lnec.pt (M.J.R.); 2 AdA—Águas do Algarve S.A., Rua do Repouso, 8000-302 Faro, Portugal; r.coelho@adp.pt (R.C.); h.lucas@adp.pt (H.L.)

**Keywords:** powdered activated carbon/coagulation/microfiltration, hybrid process, ceramic membranes, microcontaminants, pesticides, pharmaceuticals, microcystins, THMFP, protozoan cysts, viruses

## Abstract

Pressurized powdered activated carbon/coagulation/ceramic microfiltration (PAC/Alum/MF) was investigated at pilot scale for treating low turbidity and low natural organic matter (NOM) surface waters spiked with organic microcontaminants. A total of 11 trials with clarified or non-clarified waters spiked with pesticides, pharmaceutical compounds, or microcystins were conducted to assess the removal of microcontaminants, NOM (as 254 nm absorbance, A254, and dissolved organic carbon, DOC), trihalomethane formation potential (THMFP), aerobic endospores as protozoan (oo)cysts indicators, bacteriophages as viruses indicators, and regular drinking water quality parameters. PAC/(Alum)/MF achieved 75% to complete removal of total microcontaminants with 4–18 mg/L of a mesoporous PAC and 2 h contact time, with a reliable particle separation (turbidity < 0.03 NTU) and low aluminium residuals. Microcontaminants showed different amenabilities to PAC adsorption, depending on their charge, hydrophobicity (Log Kow), polar surface area and aromatic rings count. Compounds less amenable to adsorption showed higher vulnerability to NOM competition (higher A254 waters), greatly benefiting from DOC-normalized PAC dose increase. PAC/Alum/MF also attained 29–47% NOM median removal, decreasing THMFP by 26%. PAC complemented NOM removal by coagulation (+15–19%), though with no substantial improvement towards THMFP and membrane fouling. Furthermore, PAC/Alum/MF was a full barrier against aerobic endospores, and PAC dosing was crucial for ≥1.1-log reduction in bacteriophages.

## 1. Introduction

Finding resilient and cost-effective water treatment, easily adaptable to seasonal and to site-specific constraints, is crucial in the current climate uncertainty context of increasing limited water resources and challenging water quality requirements. Even optimized, conventional water treatment (coagulation/flocculation/sedimentation, filtration, chlorination) has limitations against a growing number of synthetic and natural organic microcontaminants in water sources and continues to face difficulties in ensuring disinfection with minimal (controlled) disinfection by-products’ formation [1].

Pharmaceutical compounds (PhCs), pesticides and cyanotoxins are examples of organic microcontaminants usually resistant to conventional drinking water treatment (<30% removal) [2,3,4]. Diverse PhCs with potential deleterious acute and synergistic chronic health effects have been reported in surface, groundwater and even in drinking water at ng/L to low µg/L range [2,5,6], with carbamazepine, diclofenac, propranolol, and atenolol amongst the major compounds detected [7,8]. Pesticides such as atrazine, tebuconazole, diuron and dimethoate have shown critical occurrence in several water abstraction areas worldwide [9,10,11], and recent studies revealed adverse neurobehavioral effects due to prolonged exposure to pesticide mixtures, even at permitted levels [12] and higher toxicity of some pesticide metabolites compared to their parent compounds [4]. On the other hand, the frequency and distribution of toxic cyanobacterial blooms releasing toxic metabolites, such as microcystins, and aesthetically displeasing taste and odour compounds into the drinking water sources are increasing [13,14]. An example was the 2014 *Microcystis* bloom in Toledo (OH, USA) which closed the water supply for 3 days affecting 400,000 residents [13].

Microorganisms resistant to chemical oxidation, as protozoan (oo)cysts and some viruses, also challenge conventional drinking water treatment [15]. Recent studies revealed high concentrations of protozoan (oo)cysts in the treated water of several water treatment plants in Spain [16,17], and viruses such as adenoviruses, caliciviruses, enteroviruses, and hepatitis A viruses were evaluated for potential regulation by the United States Environmental Protection Agency [18].

Powdered activated carbon (PAC) is one of the best available technologies for controlling PhCs, pesticides, and cyanotoxins in conventional water treatment plants [19,20,21]; PAC is easy to implement and simultaneously avoid the potential formation of undesired by-products with unknown toxicity (sometimes more toxic than the parent compounds). Our previous studies showed that high removal of microcontaminants may be attained by PAC conventional addition (i.e., PAC/coagulation/flocculation/sedimentation), namely 65–79% removal of total-pharmaceuticals and 73–83% of total-pesticides with 3–9 mg/L of a mesoporous PAC or with 20–24 mg/L of a microporous PAC [21]. However, PAC may affect the floc formation process under challenging conditions for coagulation to occur, such as in low turbidity waters [22], and high PAC doses may have a deleterious effect on water residual turbidity, aluminium and aerobic endospores (as protozoan cysts indicators), results which highlight the importance of having a reliable downstream filtration to retain PAC fines [21]. Viruses and protozoan (oo)cysts may be removed by conventional clarification but efficacy was shown to depend on coagulation effectiveness [23] and ability to retain fine particles and microorganisms attached to them [24]. For instance, Zhu et al. [24] showed coagulation and sedimentation inability to remove viruses attached to fine particles, still visible in the supernatant after 3-h settling.

In this challenging context, the PAC/coagulation/ceramic microfiltration (MF) hybrid process is a very promising barrier for upgrading conventional water treatment plants. PAC adsorption is expected to enhance the removal of organic microcontaminants and natural organic matter (NOM). Coagulation is expected to improve the removal of particles, cyanobacterial cells, NOM, viruses and protozoa (oo)cysts and also to control membrane fouling. Microfiltration is expected to ensure a very efficient particle separation, removing bacteria, protozoan (oo)cysts and microorganisms attached to fine particles, and enabling the use of smaller PAC particles, allowing faster adsorption kinetics and thus a better performance [14,18,24,25,26,27,28]. Ceramic membranes are potentially better candidates for PAC long-term use than polymeric ones due to their higher resistance to deterioration by biofilm growth and to surface abrasion by coarse particles circulation [29,30,31,32]. There are results showing pressurized PAC/coagulation/ceramic MF to have stable operation and a high adaptative capacity to varying water quality [29,33,34,35]. However, most research on PAC/MF to date was mainly dedicated to conventional polymeric membranes and submerged configuration [14,32]. Many studies investigating pressurized hybrid low pressure membrane processes coupled with coagulation and/or PAC in the latest years focused on membrane fouling and traditional water quality parameters [29,32,33,34,35,36,37,38] or were conducted at lab scale, sometimes with synthetic waters [14,18,30,31,39,40,41,42]. Moreover, PAC has shown to assist a ceramic MF to remove viruses to below the detection limit (1.54–2.67 log removal) in a submerged PAC-MF system [20], but PAC/MF performance for disinfection has not received much attention. Pilot studies of pressurized PAC/coagulation/MF envisaging organic microcontaminants, NOM and microorganisms removal under real scenarios, with real waters and quality variations are still scarce, particularly for surface water.

Pilot studies with pressurized PAC/ultrafiltration reported removals of sulfamethoxazole, carbamazepine and diclofenac from secondary effluent of 60–95% [43] and <30% to >80% [44] with 20 mg/L PAC, while in our previous studies with a pressurized PAC/(Alum)/ceramic MF pilot [28] we reached individual pesticides’ removals of 53% to >97% from surface water using 8–10 mg/L PAC. Overall, PAC seems to be more efficient for the adsorption of neutral hydrophobic or positively charged compounds [28,45]. For low-hydrophobicity compounds, positively charged functional groups and low surface polar area and/or high number of aromatic rings seem to act as adsorption enhancers [28,46]. As adsorption onto PAC depends on contaminant properties, water matrix, PAC properties, dose and dosing conditions (e.g., contact time, mixing conditions) [28,43,45,46], the prevalent contaminant-PAC-water interactions are site-specific and a wider range of studies is important to further support generic predictions.

This paper therefore aims at investigating pressurized PAC/coagulation/ceramic MF for treating challenging waters for drinking water production, namely low turbidity/low NOM surface waters, harder to be coagulated and prone to high PAC dosing risks (PAC fines escaping with the conventionally treated water), spiked with different mixtures of organic microcontaminants and using a pilot plant operating in a water treatment plant in Portugal.

In total, 11 trials with clarified or non-clarified waters spiked with pesticides, PhCs or microcystins were conducted to assess water quality towards microcontaminants’ and NOM removal, trihalomethane formation potential (THMFP), endospores (as protozoan (oo)cysts indicators) and bacteriophages (as virus indicators), in addition to regular drinking water quality parameters. The operational performance of this PAC/Alum/ceramic MF pilot was already approached in a previous work [36], results being used herein only when necessary to support discussion. The outcomes of this paper will help guiding water authorities and water practitioners on safe barriers for microcontaminants’ control, tracing the abilities and potential limits of this hybrid process.

## 2. Materials and Methods

### 2.1. Intake Waters

Trials were conducted with clarified (W1) and non-clarified (W2) surface waters from Alcantarilha water treatment plant (Águas do Algarve S.A.) later enriched with the organic microcontaminants targeted. As shown in Table 1, both W1 and W2 waters presented low turbidity (<4.5 NTU) and low NOM content (total organic carbon, TOC < 2.9 mgC/L) and aromaticity, the latter expressed by SUVA, the specific ultraviolet absorbance (given by absorbance at 254 nm to dissolved organic carbon ratio, SUVA = A254/DOC) ≤ 1.6 L/(mg.m). W1 exhibited more variable alkalinity and W2 showed higher turbidity, DOC (+19 to +92%) and A254 content (+20 to +167%), the latter parameter indicating a potentially higher microcontaminants-NOM competition for PAC adsorption.

### 2.2. Organic Microcontaminants

A total of 36 organic microcontaminants, comprising 22 PhCs, 10 pesticides and four microcystins (Table S1 in supplementary material), were examined under different mixtures (11 spiking trials) in two intake waters (W1 or W2). A total of 10 pesticides selected for monitoring in Portugal by the Portuguese Environment Agency were studied. The 22 pharmaceuticals were selected to cover a wide range of therapeutic classes and key physicochemical properties for adsorption, namely: structure, molar mass, charge, and hydrophobicity, the latter indicated by Log Kow, where Kow is the n-octanol/water partition coefficient. Furthermore, for the sake of cost-efficiency, the 22 pharmaceuticals selected were possible to analyse with low quantification limits (0.005–0.1 µg/L) by a single laboratory, with 1–2 injections. One of the most commonly occurring cyanotoxins in surface water reservoirs used for water supply, microcystin-LR (MC-LR) [40], was also selected and, as explained below, the mixture examined included also other three minor microcystin variants: MC-LY, MC-LW and MC-LF.

Concentrated stock solutions (2–5 mg/L, deionized water) of pesticides or PhCs (Sigma–Aldrich, Saint Louis, MO, USA) were prepared and stored in the dark at around 20 °C and stirred until the trials. For each spiking trial, a new stock solution was prepared. The compounds with low water solubility, i.e., bezafibrate and indomethacin, required a stock solution of lower concentration or pre-dilution in methanol.

The microcystins stock solution was prepared from a methanolic extract of *Microcystis aeruginosa* laboratory grown cultures. Prior to the solution preparation, the microcystin stock concentration was determined by high performance liquid chromatography with photodiode-array detection (HPLC-PDA), the necessary volume was rotary evaporated and the dry extract was dissolved in deionized water (stock solution, 1480 µg/L MC-LReq). The microcystin variants detected by HPLC-PDA were MC-LR (corresponding to 88% of the overall concentration), MC-LY, MC-LW, and MC-LF, and the overall concentration was always quantified in MC-LR equivalent concentration (µg/L MC-LReq).

On the day of the trials, the stock solutions were diluted in deionized water to 250–600 µg/L (feed solution), to allow a feasible flow rate of the peristaltic pump. The feed solution (stirred and kept in the dark) was then continuously added to the pilot feed tank (by a peristaltic pump) and mixed with the intake water (W1 or W2).

### 2.3. PAC and Coagulant

After screening tests with different PACs and a short-list of contaminants (5 PhCs, 1 pesticide, 4 microcystins and NOM) in model waters and in natural waters from Alcantarilha water treatment plant, PAC Norit SA Super (Cabot Corporation, Boston, MA, USA) was selected. PAC textural characterization was subcontracted to an external lab and was performed according to Mestre et al. [47]. This alkaline PAC (pH_pzc_ of 11.3) is highly mesoporous (53% of pore volume are mesopores), has a high surface area (1126 m^2^/g) and a relatively small particle diameter (15 µm average). A 0.4–1.5 g/L PAC slurry was prepared with dechlorinated tap water and 4–18 mg/L PAC was dosed to a stirred contact tank, providing a minimum of 2 h hydraulic retention time (2.4 h average) prior to MF filtration.

Following the long-term demonstration period conducted with waters similar to W1 and W2 [36], inline coagulant dosing was conducted for membrane fouling control during trials with non-clarified waters (W2). In those trials with W2, alum (hydrated aluminium sulphate, 2–3 mg/L Al_2_O_3_) was continuously dosed into a loop, before the membrane. Prior to the pilot trials, jar tests with feed waters similar to W2 were conducted with alum and with a high basicity (≥60%) aluminium polyhydroxychlorosulfate (WAC AB). For the low coagulant doses used during the spiking trials, no differences were found between the two coagulants in terms of residual turbidity and NOM removal. Given this, and since the potential formation of pin flocs is not an issue for Alum/MF process (the MF membrane used retains flocs > 0.1 µm), we decided by alum, a cost-effective, well known and easy to obtain coagulant. For the same reason, conventional flocculation for the flocs to grow and settle could be avoided and the more cost-effective inline coagulation option could be used.

Filtration cycles without alum dosing (W1) are hereafter referred as MF and PAC/MF and those with alum dosing (W2) as Alum/MF and PAC/Alum/MF.

### 2.4. PAC/(Alum)/MF Pilot

**Figure 1:**
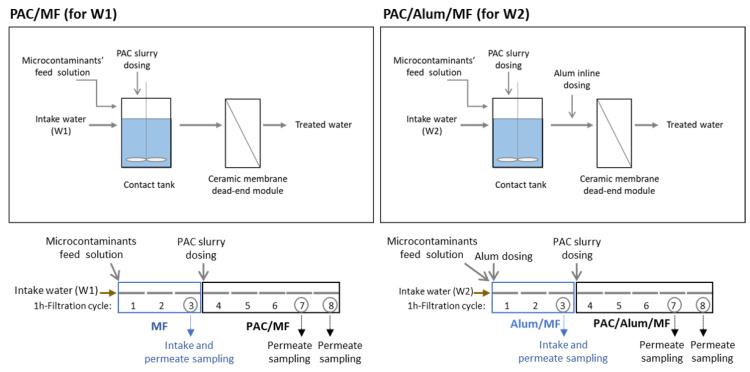
Scheme of powdered activated carbon/(coagulation)/microfiltration process, i.e., PAC/MF for intake water W1 and PAC/Alum/MF for intake water W2 (**top**) and of the respective spiking trials’ procedure (**bottom**) (8 × 1-h filtration cycles, being 3 cycles without PAC and 5 cycles with PAC dosing).

The PAC/(Alum)/MF pilot was fully automated, remote controlled and with inline monitoring of pressure, flow rate, temperature, pH and turbidity. The scheme of the PAC/(Alum)/MF process, more specifically of PAC/MF for W1 and PAC/Alum/MF for W2, is presented in Figure 1 (top). The pilot detailed scheme may be found in Campinas et al. [36].

The central component is a pressurized microfiltration module comprising three tubular MF (0.1 µm) ceramic (ZrO_2_/TiO_2_) membranes (1.2 m length and 25 mm diameter; KleanSep-Orelis, Orelis Environment SAS, Salindres, France), with 19 channels each (3.5 mm diameter each), providing a total surface area of 0.75 m^2^. During all spiking trials microfiltration was conducted in dead-end mode, at a constant flux (133 L/(m^2^·h), in short lmh), with 60-min filtration cycles followed by backwash (backwash time varied in order to always ensure 9.3 L/m^2^ at 1.4–1.5 bar). To ensure a constant flux during each 1-h filtration cycle, the transmembrane pressure varied over time depending on membrane fouling, and an average of 0.48 ± 0.07 bar (± standard deviation) was registered in the 11 spiking trials (each with eight 1-h filtration cycles).

Whereas cross-flow filtration is commonly used with polymeric membranes, ceramic membranes are often operated at dead-end filtration for it allows lower energy consumption (only for water pressurisation, not for circulation) and higher water recovery rates, provided membrane fouling is controlled and permeability easily recovered between filtration cycles. PAC/Alum/ceramic MF operational performance was earlier optimised [36]. This long-term pilot study showed dead-end filtration of waters similar to those herein used was feasible, achieving a high treatment capacity, an indicator incorporating key aspects of process productivity (membrane area, applied pressure, water volume effectively produced). The good performance of dead-end filtration results from the low fouling potential and easy cleaning of the ceramic membrane (allowing high doses of chlorine and vigorous backwash) and the water low fouling and scaling character (low NOM content and aromaticity, no algae-rich material; no high levels of iron, manganese and hardness).

PAC/(Alum)/MF pilot was designed for a long-term (1.5 years) demonstration and optimisation of this hybrid process in a Portuguese water treatment plant to demonstrate the process effectiveness, reliability and efficiency under several scenarios of water quality. At the time of pilot design, preliminary laboratory tests highlighted the importance of providing an effective contact time between PAC and the target microcontaminants preferentially above 30 min. A minimum contact time of 1 h was therefore selected, resulting in a feed tank with 240 L average capacity for the maximum membrane flux to be tested, 330 L/(m^2^·h) (0.75 m^2^ membrane area). The 11 spiking trials presented in this paper were conducted throughout the 1.5-year period and a conservative flux of 133 L/(m^2^·h), possible to be used for all waters tested, was selected, corresponding to 2.4 h average contact time (2 h minimum), which was not further subjected to optimisation. The adequate contact time for PAC/Alum/MF depends on microcontaminants’ characteristics and NOM-microcontaminants competition, some compounds benefiting with contact time increase, others not being much affected, as demonstrated in other studies including ours comparing inline vs. tank PAC dosing [28].

### 2.5. PAC/(Alum)/MF Trials

The 11 spiking trials were conducted in PAC/(Alum)/MF pilot, using different intake waters (6 spiking trials with W1 and 5 with W2), classes of organic microcontaminants (PhCs, pesticides, a mixture of both or microcystins), microcontaminants’ initial concentration (8.8–17.5 µg/L total-PhCs, 1.3–10.8 µg/L total-pesticides, 1.3 µg/L MC-LReq) and PAC doses (4–18 mg/L PAC). The conditions of the spiking trials are summarized in Table 2. The rationale behind the microcontaminant mixtures examined was to study a set of scenarios of health-environmental concern related with different drinking water origins, i.e., surface waters contaminated with: pesticides (spiking trials (Spk) 1, 4, 9, and 11, to test different operating conditions; Table 2) or toxins produced by cyanobacterial blooms (triggered by phosphorus and nitrogen; Spk 6) from agricultural runoff; with PhCs from urban treated wastewater discharges (Spk 2, 8, and 10); with PhCs and pesticides (Spk 3, 5, and 7).

Immediately before each spiking trial, a pre-determined volume of the microcontaminants’ spiking solution was supplemented to the feed tank to accelerate the intended steady-state concentration of microcontaminants. Trials begun with intake water (W1 or W2) being continuously added to the feed tank and mixed with the microcontaminants’ feed solution delivered by a peristatic pump.

Each spiking trial comprised 8 cycles of 1 h-filtration each, starting with 3 cycles without PAC addition (MF for W1 or inline Alum/MF for W2), followed by 5 cycles with PAC continuous dosing to the contact tank (PAC/MF for W1 or PAC/Alum/MF for W2) (Figure 1, bottom). At the beginning of cycle 4 (the first filtration cycle with PAC dosing), a pre-determined PAC mass was added to the contact tank for speeding up the intended steady-state PAC concentration. Afterwards, the PAC slurry was continuously dosed to the feed tank. The membrane cleaning consisted of (i) backwashing with permeate water after each 1-h filtration cycle, to remove the accumulated solids, and (ii) chemically enhanced backwashing (CEB) (with 1000 mg/L sodium hypochlorite for fouling removal or 1800 mg/L sulfuric acid for scaling removal) after each spiking trial, i.e., after 8 filtration cycles, to recover the membrane permeability and ensure the subsequent runs started with comparable filtration conditions. This procedure and the optimal 1-h filtration duration were established within the previous optimisation studies [36] to allow stable operation with the different waters tested, maximizing the treatment capacity and the fouling control.

All trials required an earlier authorization from the local Environment Agency due to microcontaminants’ spiking. The terms agreed were that the waters produced in these trials, namely the permeate waters and the backwash waters with the spent PAC, could be slowly and gradually discharged into the sludge phase of the full-scale water treatment plant (the spent PAC ultimately “diluted” in the plant’s sludge) provided low concentrations and low volumes (as low as possible for results’ representativeness) were involved. Otherwise, they would have to be collected and treated by a specialized external company. Short-term trials were therefore always conducted to reduce the amount of residues produced.

### 2.6. Sampling and Analysis

Intake samples were collected in the third filtration cycle; permeate samples were collected in the third filtration cycle (MF or Alum/MF, one sample) and in the last two filtration cycles (PAC/MF or PAC/Alum/MF, two samples) (Figure 1, bottom). With the exception of grab samples collected for microbiological analysis (aerobic endospores and bacteriophages), all samples were 5-portions composite samples (collected at 10 min, 20 min, 30 min, 40 min, and 50 min of the 1-h filtration cycles).

The analysis of PhCs, by liquid chromatography-tandem mass spectrometry (LC-MS/MS), and of pesticides, by ultra-high performance liquid chromatography coupled to tandem mass spectrometry (UPLC-MS/MS), were subcontracted to external laboratories certified for these parameters (Vitens, Holland, for PhCs; Laboratório de Análises, Instituto Superior Técnico (IST), Lisbon, for pesticides). The IST laboratory also performed the detection and quantification of bacteriophages based on the double-layer method and according to ISO (International Standardization Organization) standards: ISO 10705-1:1995 for Enumeration of F-specific RNA bacteriophages, ISO 10705-2:2000 for enumeration of somatic coliphages and ISO 10705-4:2001 for the enumeration of bacteriophages infecting Bacteroides fragilis. Microcystins and other regular water quality parameters were analysed in Águas do Algarve accredited laboratory, the former by high performance liquid chromatography with photodiode-array detection following standard operation procedures developed by Meriluoto and Spoof and described in Campinas and Rosa [40], and the latter using standard methods for the examination of water and wastewater (SMEWW) [48]. Turbidity was measured by nephelometry (ISO 7027-1:2016), TOC and DOC by high temperature combustion with infrared detector (EN 1484:1997), A254 by UV–VIS spectrophotometry (SMEWW 5910 B, using quartz cells with 50 mm optical path length), total aluminium by molecular absorption spectrophotometry (ASTM D3919:2015, from the American Society for Testing and Materials) and alkalinity by potentiometric titration (SMEWW 2320 B). DOC and A254 were measured on samples pre-filtered through 0.45-µm membrane filters. THMFP was measured by a simplified method adapted from SMEWW [48]. The aerobic endospores were herein used as indicators of protozoan (oo)cysts removal and were also analysed by Águas do Algarve by enumeration after heat treatment to inactivate any vegetative cells, filtering and aerobic incubation at 35 °C.

### 2.7. Statistical Methods

The statistical significance (*p*-values) of differences in pilot intake median concentrations for NOM and THMFP or in NOM median removal efficiencies and THMFP median reduction in the four different configurations tested (MF, PAC/MF, Alum/MF, and PAC/Alum/MF) was assessed through statistical tests using the Past 4.01 program. Similar tests were also used to assess microcontaminants’ charge influence on their removal. PhCs were grouped according with their charge, in positively charged, neutral and negatively charged, and statistical tests were conducted to assess the statistical significance (*p*-values) of median removal efficiency differences between groups. Briefly, one-way ANOVA (homogeneous variance with Levene’s test) or Welch F test (unequal variance) were conducted for normal distributions, and Kruskal Wallis test was used for non-normal distributions (Shapiro-Wilk test, *p*-values < 0.05). Significance levels of 0.1 were applied instead of the usual 0.05 due to the low sample size [21,49].

## 3. Results and Discussion

### 3.1. Turbidity Removal and Aluminium Residuals

PAC particle retention is crucial for an effective removal of microcontaminants, but in low-turbidity waters as those studied, there is an intrinsic coagulation difficulty due to the limiting rate of inter-particle contacts, and PAC has shown to add to this difficulty by affecting the floc formation process [22]. In our recent work with PAC/coagulation/flocculation/sedimentation [21], we observed that PAC dosing, particularly above 10 mg/L PAC, hampered the clarification of the low-turbidity waters studied towards residual turbidity, aluminium and aerobic endospores, and could require a reliable downstream filtration to retain PAC fines. Inline permeate turbidity values in PAC/(Alum)/MF pilot were always 0.01–0.03 NTU, in all trials regardless of the intake water quality and PAC dosing. This was expected due to the MF pore size (0.1 µm), which effectively retain particles, even the small PAC fines.

Aluminium residuals in MF permeate were only measured during the spiking trials with alum addition, i.e., in trials with W2 (spiking 8 to 11). Total Al < 35 ± 28 µg/L (average ± standard deviation) was observed, far below the drinking water quality standard of 200 µg/L and much lower than the residuals obtained after treating similar low turbidity and low NOM surface waters with PAC conventional addition (PAC/coagulation/flocculation/sedimentation) [21]. In those experiments, a high contribution of particulate and colloidal material to the aluminium residuals was observed, which may explain the low Al residuals after microfiltration. In addition, with membrane hybrid processes there is a lower coagulant demand for an effective particle separation. Results with PAC/(Alum)/ceramic MF confirm this process as a reliable option for effectively removing turbidity while maintaining low aluminium residuals.

### 3.2. PhCs, Pesticides, and Microcystins Removal

**Figure 2:**
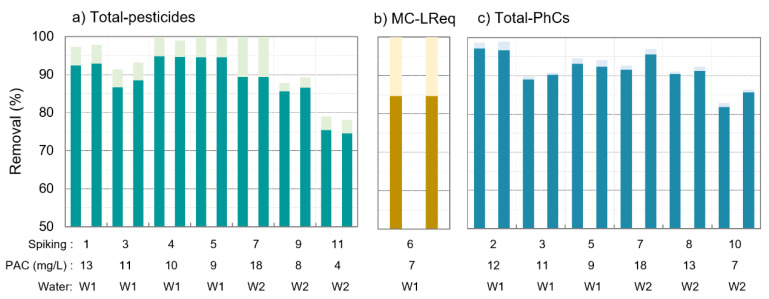
Removal of total-pesticides (**a**), microcystin-LR equivalent (**b**) and total-pharmaceuticals (**c**) by PAC/(Alum)/MF in each of the 11 spiking trials (2 samples per spiking; due to some microcontaminants’ concentrations < limit of quantification (LOQ), removals ranged between the values computed with LOQ for those microcontaminants, represented in darker colours, and values computed with 0 µg/L, in lighter colours).

The removal of total-pesticides, microcystins (MC-LReq), and total-PhCs after PAC/(Alum)/MF is presented in Figure 2 (2 samples per spiking trial), where the total-pesticides and total-PhCs concentrations are given by summing the concentrations of all pesticides or PhCs analysed. When a compound’s concentration in the permeate water was below the limit of quantification (LOQ), the removal efficiency varied between the value considering for that contaminant its LOQ (darker colours) and the value considering 0 µg/L (lighter colours).

PAC/(Alum)/MF achieved high microcontaminants’ removal, between 75% and complete removal (final concentration below LOQ) of total-pesticides with 4–18 mg/L PAC (Figure 2a), 85% to complete removal of microcystin-LReq (all microcystin variants had concentrations < LOQ) with 7 mg/L PAC (Figure 2b) and 82–98% for total-PhCs with 7–18 mg/L PAC (Figure 2c). As the coagulant has shown in our previous studies with PAC/coagulation/flocculation/sedimentation [21] to have no effect on microcontaminants’ removal from waters similar to those herein used, we may assume microcontaminants’ removal by PAC/(Alum)/MF is mostly due to PAC adsorption. Total-pesticides and total-PhCs removal values with PAC/(Alum)/MF were relatively similar to those verified with PAC conventional addition [21], with differences below 7% for equivalent waters (W2) and PAC doses (3–7 mg/L PAC). One substantial advantage for the hybrid membrane process is nevertheless the total retention of PAC fines and the low aluminium residuals, as discussed in Section 3.1.

**Figure 3:**
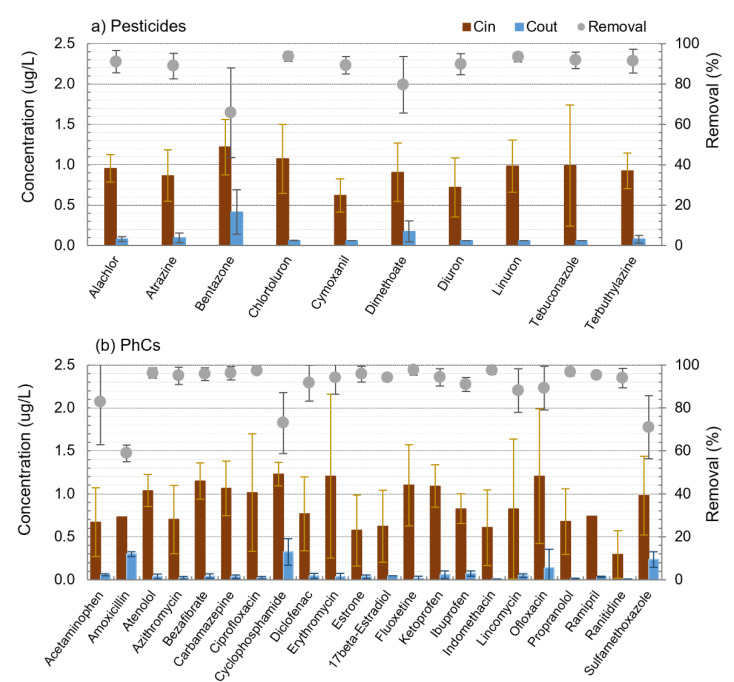
Average intake (brown bars) and permeate (blue bars) concentrations and removals (circles) of pesticides (**a**) and pharmaceuticals (**b**) after PAC/(Alum)/MF (average values of all spiking trials; error bars represent standard deviations between trials).

The average intake concentrations for individual pesticides and PhCs are depicted in Figure 3 together with the average concentrations and removals after PAC/(Alum)/MF (average values for all spiking trials), where error bars represent standard deviations between trials. Higher error bars point to higher vulnerability of the compound to the trial conditions. As expected, individual pesticides presented different PAC adsorption behaviours (Figure 3a). Excluding bentazone and dimethoate, all pesticides showed high removal values (average >89%, 76% minimum, >97% maximum) and low removal variability between trials, with 2–6% standard deviations for all conditions tested. Dimethoate and bentazone were more vulnerable to trial conditions, with removals varying between 53 and >93% for the former and 35–88% for the latter, their average permeate concentrations being above the Drinking Water Directive limit (EU 2020/2184) of 0.1 µg/L.

Overall, high removals were also observed for PhCs (Figure 3b), with 14 compounds out of 22 presenting minimum removal above 85% and 19 out of 22 with average removals also above that value. Similarly to pesticides, individual PhCs also presented different PAC adsorption behaviours. Some compounds had always removals ≥ 90%, the case of atenolol, azithromycin, bezafibrate, ciprofloxacin, estrone, beta-estradiol, fluoxetine, and propranolol, while others presented lower removal or higher removal variability between trials, namely acetaminophen (83 ± 20%), amoxicillin (59 ± 4%), cyclophosphamide (73 ± 14%), and sulfamethoxazole (71 ± 15%).

**Figure 4:**
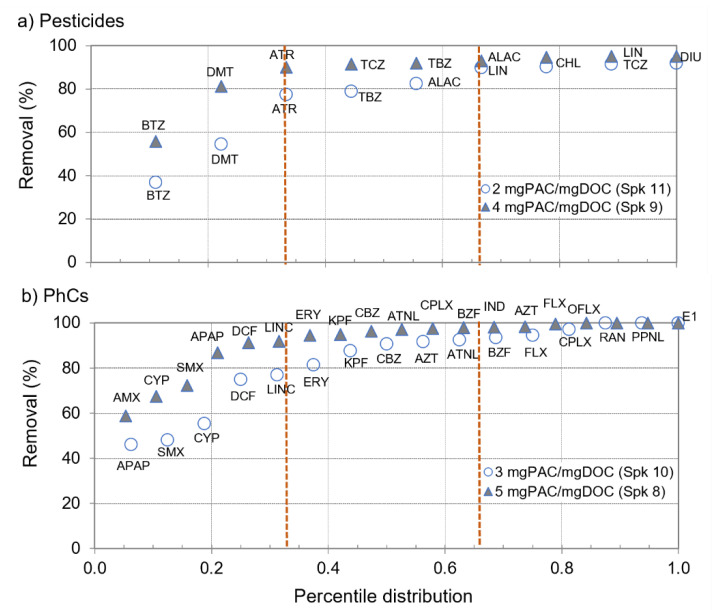
Percentile distribution of removal efficiencies of 9 pesticides (**a**) and 16–19 PhCs (**b**) during two spiking trials each (those with the highest number of microcontaminants and the lowest PAC doses).

To analyse the microcontaminants’ amenability to PAC adsorption the percentile distribution of removal efficiencies of pesticides and PhCs during two spiking trials (those with the highest number of microcontaminants and the lowest PAC doses, i.e., spiking 9 and 11 for pesticides and spiking 8 and 10 for PhCs, Table 2) is represented in Figure 4. The compounds were then grouped in three percentile ranges for further analysis: P33 (less amenable); P34–P67; >P67 (more amenable).

**Figure 5:**
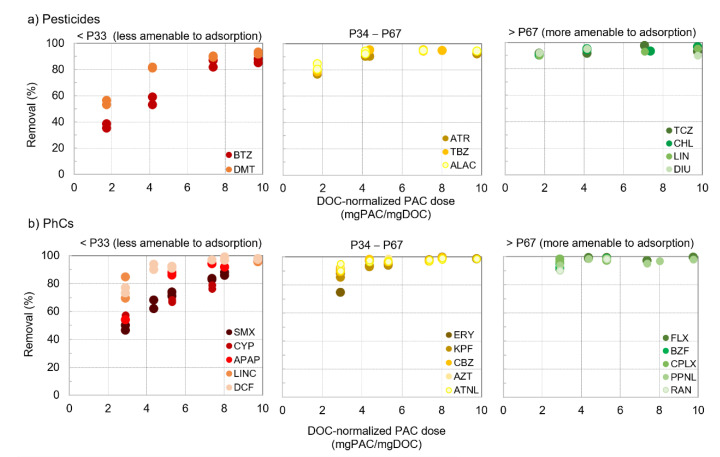
Removal as a function of DOC-normalized PAC dose for a pool of individual pesticides (**a**) and PhCs (**b**) presenting different amenabilities to PAC adsorption.

Except for minor differences, results were very consistent with those previously obtained by PAC/coagulation/flocculation/sedimentation with an alike pool of microcontaminants in similar waters (W2), though with a different PAC (Sorbopor MV12) [21]: ofloxacin and alachlor showed lower amenability to adsorption in that work, and the opposite occurred for tebuconazole and diclofenac. Figure 4 suggests a different impact of DOC-normalized PAC dose on compounds with different amenabilities to PAC adsorption. To further explore this aspect, the removals as a function of DOC-normalized PAC doses (PAC/DOC ratio, mg PAC/mg DOC) were compared for some pesticides and PhCs (Figure 5).

It is clearly observed that an increase in DOC-normalized PAC dose mostly benefits the removal of compounds less amenable to adsorption, its impact decreasing with the compounds’ amenability up to almost no effect for those more amenable to adsorption (Figure 5). For instance, the pesticide bentazone (BTZ, Figure 5a left) showed 35–38% removal with 1.7 mg PAC/mg DOC, increasing to 53–59% with 4.2 mg PAC/mg DOC and to 82–87% with 7.4 mg PAC/mg DOC. On the contrary, the PhCs fluoxetine (FLX, Figure 5b right) showed 91–98% removal with 2.9 mg PAC/mg DOC, increasing to 98–99% with 4.4 mg PAC/mg DOC and to >97% with 7.4 mg PAC/mg DOC.

**Figure 6:**
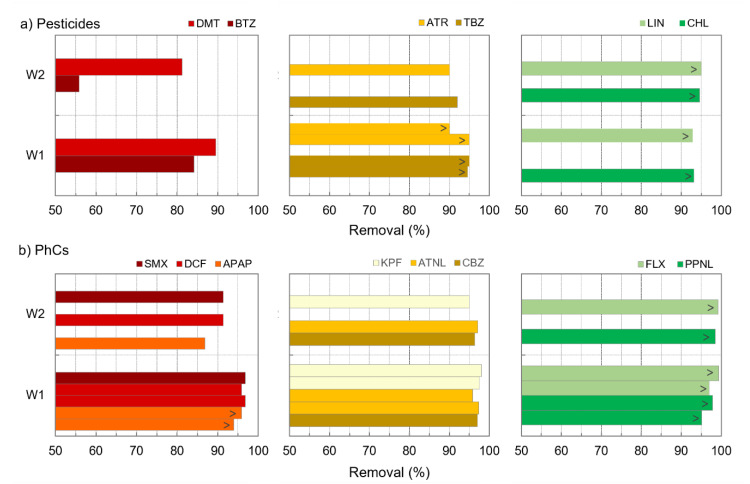
Removal of individual pesticides (**a**) and PhCs (**b**) presenting different amenabilities to PAC adsorption as a function of intake water, W1 or W2 (trials with similar PAC doses and different intake waters were compared; 8–11 mg/L PAC for pesticides and 11–13 mg/L PAC for PhCs; “>”for removals computed with LOQ).

**Figure 7:**
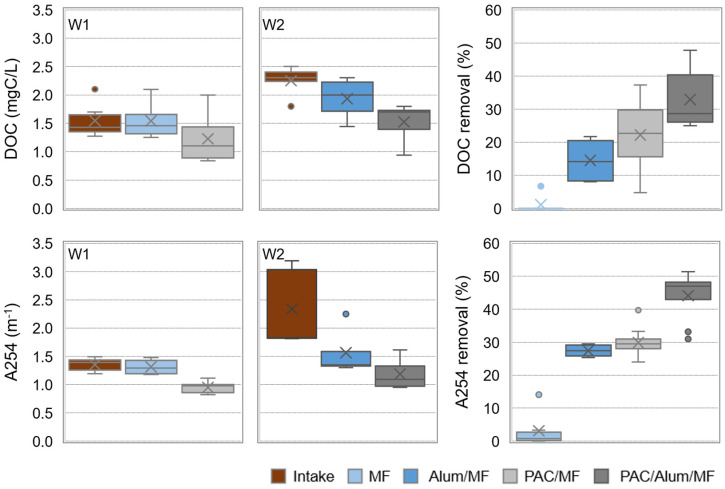
NOM content, as DOC and A254, in pilot intake (W1 or W2) and permeate waters after MF and PAC/MF (for W1, 7–13 mg/L PAC) or Alum/MF and PAC/Alum/MF (for W2, 2–3 mg/L Al_2_O_3_ and 4–13 mg/L PAC) and respective removals (box plots with maximum, P75, average, median, P25 and minimum values; little circles are outliers).

Compounds less amenable to adsorption were also more vulnerable to NOM competition, which seems evident from their lower removals from W2 compared to W1 for similar PAC doses (Figure 6). For instance, the pesticides dimethoate and bentazone (DMT and BTZ, Figure 6a left) and the PhCs sulfamethoxazole, diclofenac and acetaminophen (SMX, DCF, and APAP, Figure 6b left) showed +6 to +28% additional removal in W1 compared to W2, while such difference was reduced up to +10% for compounds such as atrazine, terbuthylazine, ketoprofen, atenolol, and carbamazepine (Figure 6a,b, middle) and no difference was observed for highly adsorbed compounds such as linuron, diuron, fluoxetine, and propranolol (Figure 6a,b, right). Despite both W1 and W2 have low NOM content and aromaticity, they possess DOC and A254 content significantly different (*p*-values < 0.005), with P75 of 1.6 mgC/L (W1) vs. 2.4 mgC/L (W2) for DOC and 1.4 m^−1^ (W1) vs. 3.0 m^−1^ (W2) for A254 (evident in Figure 7, Section 3.3), indicating a potentially higher microcontaminants-NOM competition for PAC adsorption in W2.

To assess microcontaminants’ charge influence on their removal, PhCs were grouped in positively charged, neutral and negatively charged (Table S1 in supplementary material), and statistical tests were conducted to assess the statistical significance (*p*-values) of median removal efficiency differences between groups (Table S2 in supplementary material). In spite of the positive net charge of PAC SA Super at the waters’ pH, the positively charged compounds were significantly better removed than the negatively charged ones in all, except one (spiking 2), spiking trials (*p*-values < 0.09). This is particularly noticeable by the lower end removal efficiencies per charge group, with values usually > 92% for positively charged compounds and between 46–87% for negatively charged compounds (Table S3 in supplementary material). These results are coherent with other studies’ conclusions [3,21,45], but herein with a more notorious effect if one compares them with the results of the positively charged PAC used in our previous study [21]. Furthermore, overall, the conclusions previously established for PAC conventional application [21] were herein also verified, namely the better adsorption of positively charged compounds with low polar surface area and high number of aromatic rings, and of neutral compounds with high hydrophobicity (Log Kow) and high number of aromatic rings (Figure S1 in supplementary material). The adsorption of negatively charged microcontaminants was apparently better for those presenting high Log Kow, high aromatic rings count and low polar surface area.

### 3.3. NOM Removal and Trihalomethane Formation Potential (THMFP) Reduction

**Figure 8:**
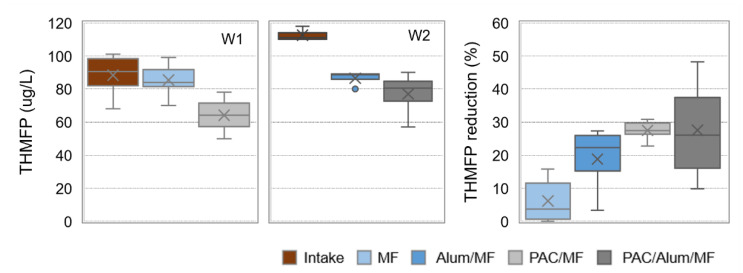
Trihalomethane formation potential in pilot intake (W1 and W2) and permeate water after MF and PAC/MF (for W1, 7–13 mg/L PAC) or Alum/MF and PAC/Alum/MF (for W2, 2–3 mg/L Al_2_O_3_ and 4–13 mg/L PAC) and respective removals (box plots with maximum, P75, average, median, P25 and minimum values; little circles are outliers).

The DOC and A254 contents in pilot intake and in permeate water after MF and PAC/MF (for W1) or Alum/MF and PAC/Alum/MF (for W2) are depicted in Figure 7, as well as the respective removals. Box plots represent maximum, P75, average, median, P25 and minimum values. Similar box plots are also presented for trihalomethane formation potential (THMFP) in Figure 8.

Microfiltration alone did not result in effective DOC and A254 removal from W1 (Figure 7), nor in THMFP reduction (4% median, Figure 8). When 7–13 mg/L PAC was added, DOC and A254 median removal significantly enhanced (*p*-values < 0.001) to 23 and 30%, respectively, and THMFP reduction also showed a significant increase (*p*-value of 5.1 × 10^–5^) to 27% median (MF and PAC/MF, Figure 7 and Figure 8).

On the other hand, microfiltration combined with inline coagulation (Alum/MF) was able to decrease the NOM content from the non-clarified waters (W2), though with a greater and more significant impact on A254 removal (28% median, *p*-value of 0.08) than on DOC removal (14% median, *p*-value of 0.2). This NOM decrease is important for THMFP decline—a median reduction of 22% is observed with Alum/MF (Figure 8)—but also for controlling backwashable and chemically reversible (by chemically enhanced backwashing) membrane fouling. This latter conclusion is supported by operational results from a previous study with the same pilot and non-clarified waters similar to W2 [36], which showed that inline alum coagulation almost doubled the treatment capacity, an indicator incorporating key aspects of process productivity, in comparison with no coagulant addition. Most studies suggest that the high molar mass biopolymer fraction of NOM is primarily responsible for irreversible membrane fouling and that coagulation, by substantially removing that fraction, effectively controls colloidal fouling (i.e., pore blockage) [29]. When 4–13 mg/L PAC was added to Alum/MF, NOM median removal was further increased (*p*-values < 0.06) to 47% for A254 and to 29% for DOC (Alum/MF and PAC/Alum/MF in Figure 7). Despite this NOM removal enhancement, the improvement of THMFP reduction and the membrane fouling control were not substantial when comparing PAC/Alum/MF with Alum/MF. In fact, THMFP median reduction with PAC/Alum/MF was 26%, statistically not different (*p*-value of 0.4) from the value registered with Alum/MF. Furthermore, the operational results from our previous study [36] showed 6–24 mg/L PAC dosing had a minimal effect on membrane chemically reversible fouling, keeping treatment capacity constant or slightly increasing it. Results are consistent with studies showing coagulation and PAC adsorption to complement each other in DOC removal, both preferentially removing hydrophobic and A254 substances, with coagulation acting preferentially on high molar mass NOM and PAC adsorption on low molar mass NOM [29,39,50]. Therefore, PAC relevance to membrane fouling control depends on low molar mass NOM contribution to fouling, coagulation usually assuming a more important role by controlling colloidal fouling.

**Figure 9:**
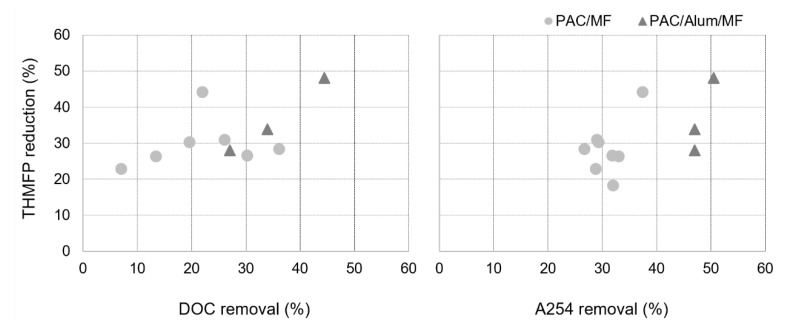
Trihalomethane formation potential (THMFP) reduction by PAC/MF and PAC/Alum/MF and NOM removal in terms of DOC (**left**) or A254 (**right**).

A good correlation was observed between A254 removal and THMFP reduction (R^2^ = 0.92) (Figure 9 right) and between DOC removal and THMFP reduction (R^2^ = 0.99) for PAC/Alum/MF (Figure 9 left), though not for PAC/MF. Coherently with our PAC/Alum/MF results, a recent study with data from 30 water treatment plants across Scotland over a 30-month period showed A254, DOC and DOC hydrophobic fraction to be good indicators of THMFP (R^2^ = 0.79–0.82) and haloacetic formation potential (R^2^ = 0.71–0.73) [51]. The higher A254 values in W2 (1.8–3.2 m^−1^ for W2 vs. 1.2–1.5 m^−1^ for W1, minimum–maximum values) also justify its significantly higher THMFP (*p*-value of 0.04), namely 111 µg/L in W2 vs. 91 µg/L in W1.

### 3.4. Bacteriophages (as Virus Indicators) and Aerobic Endospores (as Protozoan (oo)Cysts Indicators) Removal

**Figure 10:**
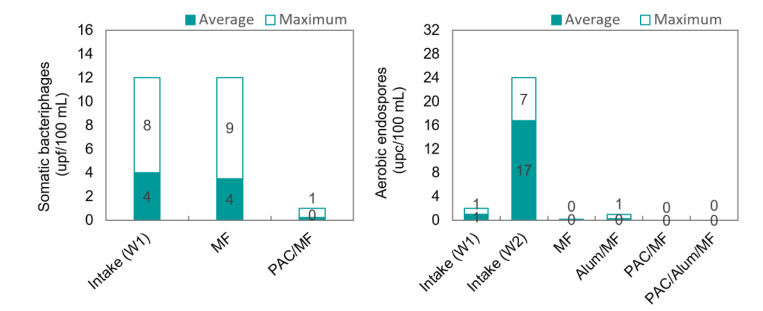
Somatic bacteriophages (**left**) and aerobic endospores (**right**) average and maximum values in pilot intake and permeate waters after ceramic MF, Alum/MF, PAC/MF and PAC/Alum/MF.

Bacteriophages, i.e., viruses that infect bacterial cells, are non-toxic and non-pathogenic for humans, animals, or plants, are reasonably similar to mammalian viral pathogens in size, shape, morphology, but are easier and less expensive to isolate and enumerate relative to enteric viruses, which explains their use as surrogates for pathogenic virus removal performance in several studies [18,24,52]. Generally, three bacteriophage groups, namely somatic coliphages, male-specific F-RNA phages and Bacteroides fragilis phages, are frequently used as surrogates for pathogenic viruses in environmental studies [52], and were monitored during trials. Positive results were only verified for somatic bacteriophages in W1 intake, meaning that only MF and PAC/MF could be assessed for their removal (Figure 10).

The similar bacteriophage concentrations in pilot intake and MF permeate (Figure 10, left) confirm that the ceramic MF membrane cannot act as a barrier to viruses (<0.3-log removal), which was expected as viruses (0.02–0.1 µm [24]) are much smaller than MF pores. On the contrary, dosing 12–13 mg/L PAC before MF decreased bacteriophage concentrations to 0–1 ufp/100 mL. Although PAC/MF may not be considered a virus full barrier, PAC dosing was crucial for attaining ≥ 1.1-log reduction. Virus removal by PAC/MF may occur due to electrostatic interactions between the negatively charged virus particles and the positively charged PAC or by virus retention in the cake layer resulting from PAC-NOM deposition on membrane surface. It is well accepted that viruses can be adsorbed onto positively charged particles, e.g., onto iron or aluminium hydroxide flocs, and virus removal has been shown to improve with higher feed turbidity [24]. Virus adsorption onto a cake layer resulting from the accumulation of large-sized organic matter on top of membranes was also proposed as a virus rejection mechanism for low-pressure membranes [53]. Alum/MF and PAC/Alum/MF could not be assessed for virus removal in our trials (no positive results in W2 intake), but, according with literature, high virus removal may be achieved by coagulation/MF, much depending on the coagulant concentration used [24]. For instance, Shirasaki et al. [18] reported 4-log reduction in bacteriophages with 0.5 mgAl/L and 6-log reduction with 1.1 mgAl/L and Zhu et al. [24] reported almost 4-log removal with 10 mgFe/L of ferric chloride (pH 7.3–8.3) and around 1-log removal with half the coagulant dose. Given the positive effect of both PAC and coagulant on virus removal, a synergistic effect is therefore anticipated, with a high potential for the hybrid PAC/coagulation/MF process, but further tests should be conducted to confirm it.

As the routine monitoring of *Cryptosporidium* and *Giardia* (oo)cysts was restricted due to financial and methodological constraints (for instance, a great volume of sampling water, around 100 L, would be necessary to be concentrated in a cartridge), it was decided to use the spores of aerobic spore-forming bacteria (aerobic endospores) as protozoan cysts indicators. Aerobic spores and oocysts share many commonalities with regard to biology and survivability, and have been suggested as a promising surrogate for *Cryptosporidium* oocysts in surface and groundwater, including by the United States Environmental Protection Agency [54].

Aerobic endospore concentrations in pilot intake and permeate waters after ceramic MF, Alum/MF, PAC/MF and PAC/Alum/MF are depicted in Figure 10 (right). Average endospore values of 1 ufc/100 mL were registered for W1 intake (2 ufc/100 mL maximum) and 17 ufc/100 mL for W2 intake (24 ufc/100 mL maximum). After MF, endospores were detected in only one sample, during one Alum/MF filtration cycle with W2, and at very low concentration (1 ufc/100 mL), never being detected in PAC/(Alum)/MF filtration cycles. Our results and the fact that endospores should be a conservative indicator for protozoan oo(cysts), since they are much smaller in size (0.8–2.0 µm) than *Giardia* cysts (8–13 µm) or *Cryptosporidium* oocysts (4–6 µm) [54] and closer to microfiltration pore size, show PAC/Alum/MF as an effective barrier against protozoan (oo)cysts.

## 4. Conclusions

PAC/(Alum)/MF achieved high microcontaminants’ removal, between >75% and >97% for total-pesticides, microcystin-LReq and total-pharmaceuticals with 4–18 mg/L of a mesoporous PAC and 2 h minimum contact time. Microcontaminants showed different amenabilities to PAC adsorption, much depending on solute key properties, such as charge, hydrophobicity (Log Kow), polar surface area, and aromatic rings count. Compounds less amenable to adsorption showed higher vulnerability to NOM competition, more expressive in non-clarified waters presenting higher absorbance at 254 nm, and benefited the most with the increase in DOC-normalized PAC dose.

PAC/Alum/MF proved to be a reliable option for effectively removing turbidity (<0.03 NTU) while maintaining low residuals of aluminium, an important advantage over PAC conventional application which, in previous works, has shown to affect clarification of low-turbidity waters towards residual turbidity, aluminium, and aerobic endospores.

Both PAC/MF and Alum/MF attained a considerable removal of natural organic matter (14–30%, median), which revealed to be important for reducing the trihalomethane formation potential (22–27%, median) and, in the case of Alum/MF, also important for controlling membrane fouling. Coherently with other studies, absorbance at 254 nm removal revealed to be a good indicator of trihalomethane formation potential reduction. Moreover, PAC complemented coagulation, adding 15–19% to NOM median removal, whereas no substantial improvement was observed for trihalomethane formation potential reduction and membrane fouling control. As PAC mainly adsorbs low molar mass compounds, further tests should be conducted to evaluate its ability for decreasing assimilable organic carbon and subsequent potential benefit on water stability in the distribution system.

PAC/Alum/MF was effective for removing microorganisms resistant to conventional chemical oxidation, being a full barrier against aerobic endospores, indicators of protozoan (oo)cysts. Though PAC/MF was not a full barrier against viruses, PAC dosing was crucial for removing bacteriophages, indicators for viruses (≥ 1.1-log reduction). Based on our PAC/MF results and on Alum/MF literature, PAC and Alum synergistic positive effects onto virus removal are expected on PAC/Alum/MF, though further tests are necessary to confirm it.

Overall, PAC/(Alum)/MF is a good option for treating challenging waters for drinking water production, providing a simultaneous effective control of organic microcontaminants, turbidity and aluminium residuals, trihalomethane formation potential, viruses, protozoan (oo)cysts and membrane fouling. It was also clear that higher PAC doses than those herein used or a multi-barrier treatment approach might be advisable if highly challenging waters or conditions are expected to be found in drinking water production, such as high concentrations of compounds with low amenability to adsorption or with high A254-absorbing NOM of relatively low molar mass. Further investigation should be conducted in this area.

## Figures and Tables

**Table 1:** Characteristics of PAC/coagulation/MF pilot intake waters (average ± standard deviation).

Water	T (°C)	pH	Alkalinity (mg/L CaCO_3_)	Turbidity (NTU)	TOC (mgC/L)	DOC (mgC/L)	A254 (m^−1^)	SUVA (L/(mg·m))
W1	18 ± 2	7.6 ± 0.1	81 ± 23	0.6 ± 0.3	1.6 ± 0.3	1.5 ± 0.3	1.3 ± 0.1	1.0 ± 0.2
W2	24 ± 4	7.7 ± 0.1	62 ± 5	3.0 ± 1.5	2.5 ± 0.4	2.2 ± 0.3	2.3 ± 0.7	1.1 ± 0.5

**Table 2:** Summary of the eleven spiking trials conducted in PAC/(Alum)/MF pilot.

Spiking Trial	Intake Water	Microcontaminant Spiked	Microcontaminants’ Initial Concentration, µg/L (Total)			PAC	Alum
			PhCs	Pesticides	MC-LReq	mg/L	mg/L Al_2_O_3_
Spk 1	W1	10 pesticides		9.9		13	0
Spk 2	W1	14 PhCs	17.5			12	0
Spk3	W1	13 PhCs + 5 pesticides	9.1	3.8		11	0
Spk 4	W1	6 pesticides		8.4		10	0
Spk 5	W1	10 PhCs + 3 pesticides	10.4	1.3		9	0
Spk 6	W1	4 microcystins			1.3	7	0
Spk 7	W2	11 PhCs + 2 pesticides	11.3	1.7		18	0
Spk 8	W2	19 PhCs	17.5			13	2
Spk 9	W2	10 pesticides		10.8		8	3.1
Spk 10	W2	16 PhCs	8.8			7	2
Spk 11	W2	9 pesticides		6.9		4	2

## Supplementary Materials

The following are available online at https://www.mdpi.com/2077-0375/11/2/91/s1, Figure S1. Pharmaceuticals’ removal with PAC/(Alum)/MF (spikings 8 and 10) vs. their hydrophobicity (measured by Log Kow) (left), aromatic ring count (middle) or polar surface area (right), Table S1. Properties of the studied organic microcontaminants (data from Chemspider/ChemAxon, last access in Jan 2020); Table S2. *p*-values for assessing statistical differences between the removal efficiencies of positively charged (+), neutral (o) and negatively charged (−) pharmaceuticals during spiking trials 2, 3, 5, 7, 8 and 10 (significant if *p*-values ≤ 0.1); Table S3. Minimum, median and maximum values of the removal efficiencies for positively charged (+), neutral (0) and negatively charged (−) pharmaceuticals during spiking trials 2, 3, 5, 7, 8 and 10 (when the compound’s concentration was below the limit of quantification (LOQ), the removal efficiency was considered to be 100%).
