# Supplementary material for: Adsorption/Coagulation/Ceramic Microfiltration for Treating Challenging Waters for Drinking Water Production
Source: Membranes (Basel). 2021 Jan 27;11(2):91. doi: 10.3390/membranes11020091 (PMC7911376; doi:10.3390/membranes11020091)
Supplement: Supplementary file 1 [file membranes-11-00091-s001.pdf]

membranes MDPI

Supplementary file

Adsorption/Coagulation/Ceramic Microfiltration for Treating Challenging Waters for Drinking Water Production

Margarida Campinas [1,*], Rui M.C. Viegas [1], Rosário Coelho [2], Helena Lucas [2] and Maria João Rosa [1]

[1] Water Quality and Treatment Laboratory, Urban Water Unit, Hydraulics and Environment Department, LNEC—National Civil Engineering Laboratory, 1700-066 Lisbon, Portugal; mcampinas@lnec.pt; rviegas@lnec.pt (R.M.C.V.); mjrosa@lnec.pt (M.J.R.)
[2] AdA—Águas do Algarve S.A., Rua do Repouso, 8000-302 Faro, Portugal; r.coelho@adp.pt (R.C.); h.lucas@adp.pt (H.L.)
* Correspondence: mcampinas@lnec.pt

Table S1. Properties of the studied organic microcontaminants (data from Chemspider/ChemAxon, last access in Jan 2020).

Charge at pH 7.8	Compound		Category	Molar mass (Da)	Log Kow	Log D at pH 7.8	PSA* Å²	Ar-rings** Number
+1	Atenolol	ATNL	Beta-blocker	266	0.425	-1.43	85	1
+2	Azithromycin	AZT	Antibiotic	749	2.44	-0.46	180	0
+1	Erythromycin	ERY	Antibiotic	734	2.6	1.37	194	0
+1	Fluoxetine	FLX	Psychiatric drug	309	4.2	2.2	21	2
+1; 0 [a]	Lincomycin	LINC	Antibiotic	406	-0.32	-0.71	148	0
+1	Propranolol	PPNL	Beta-blocker	259	2.58	0.73	41	2
+1; 0 [b]	Ranitidine	RAN	Anti-ulcer	314	0.99	0.69	112	1
0	Acetaminophen	APAP	Analgesic	151	0.91	0.9	49	1
0	Carbamazepine	CBZ	Psychiatric drug	236	2.77	2.77	47	2
0 (-1) [c]	Ciprofloxacin	CPLX	Antibiotic	331	-0.86	-0.89	73	2
0	Cyclophosphamide	CYP	Antineoplastic agent	261	0.097	0.097	51	0
+1; 0 [b]	Ranitidine	RAN	Anti-ulcer	314	0.99	0.69	112	1
0	Estrone	E1	Hormone	270	4.3	4.3	37	1
0	β-Estradiol	E2	Hormone	272	3.75	3.74		1
0	Alachlor	ALAC	Pesticide	270	3.59	3.59	30	1
0	Atrazine	ATR	Pesticide	216	2.2	2.2	63	1
0	Chlortoluron	CHL	Pesticide	213	2.44	2.44	32	1
0	Cymoxanil	CYM	Pesticide	198	0.07	0.07	104	0
0	Dimethoate	DMT	Pesticide	229	0.34	0.34	118	0
0	Diuron	DIU	Pesticide	233	2.53	2.53	36	1
0	Linuron	LIN	Pesticide	249	2.3	2.3	45	1
0	Tebuconazole	TCZ	Pesticide	308	3.69	3.69	51	2
0	Terbuthylazine	TBZ	Pesticide	230	2.48	2.48	63	1
-1 (0) [d]	Amoxicillin	AMX	Antibiotic	365	-2.3	-2.9	158	1
-1	Bezafibrate	BZF	Lipid regulator	362	3.99	0.59	76	2
-1	Diclofenac	DCF	Anti-inflammatory	296	4.26	0.91	49	2
-1	Ibuprofen	IBUP	Anti-inflammatory	206	3.84	1	37	1
-1	Indomethacin	IND	Anti-inflammatory	358	3.53	0.12	69	3
-1	Ketoprofen	KPF	Anti-inflammatory	254	3.6	0.23	54	2
-1 (0) [e]	Ofloxacin	OFLX	Antibiotic	361	0.09	-0.8	73	2
-1	Ramipril	RAM	Anti-hypertensive	416	1.47	-0.32	96	1
-1	Sulfamethoxazole	SMX	Antibiotic	253	0.79	-0.08	107	2
-1	Bentazone	BTZ	Pesticide	240	0.76	-0.19	75	1
-1	Microcystin-LR	MC-LR	Cyanotoxin	995	-1.4	-4.5	341	1

* Polar surface area; ** Aromatic rings count
[a] 60% positively charged species, 40% neutral species
[b] 50% neutral species (with positively and negatively charged functional groups), 50% positively charged species
[c] 90% neutral species (with positively and negatively charged functional groups), 10% negatively charged species
[d] 77% negatively charged species, 20% neutral species (with positively and negatively charged functional groups)
[e] 92% negatively charged species, 8% neutral species (with positively and negatively charged functional groups)

Table S2. p-values for assessing statistical differences between the removal efficiencies of positively charged (+), neutral (o) and negatively charged (−) pharmaceuticals during spiking trials 2, 3, 5, 7, 8 and 10 (significant if *p*- values ≤ 0.1).

PhC	Spk 2			Spk 3			Spk 5			Spk 7			Spk 8			Spk 10		
charge	+	0	−	+	0	−	+	0	−	+	0	−	+	0	−	+	0	−
+		-	0.3		0.02	0.01		0.5	0.002		0.4	0.09		0.4	0.08		0.8	0.04
0			-			0.9			0.004			0.09			0.5			0.2
-																		

Significant difference with *p*-value ≤ 0.1 (in bold).

Table S3. Minimum, median and maximum values of the removal efficiencies for positively charged (+), neutral (0) and negatively charged (−) pharmaceuticals during spiking trials 2, 3, 5, 7, 8 and 10 (when the compound's concentration was below the limit of quantification (LOQ), the removal efficiency was considered to be 100%).

	Spk 2			Spk 3			Spk 5			Spk 7			Spk 8			Spk 10		
	+	0	−	+	0	−	+	0	−	+	0	−	+	0	−	+	0	−
**Min**	95	100	87	97	76	80	96	90	62	97	95	69	92	67	56	69	38	46
**Median**	99	-	98	100	93	94	98	99	91	98	99	93	98	97	95	92	94	81
**Max**	100	100	100	100	100	100	99	100	94	100	100	100	100	100	100	100	100	95

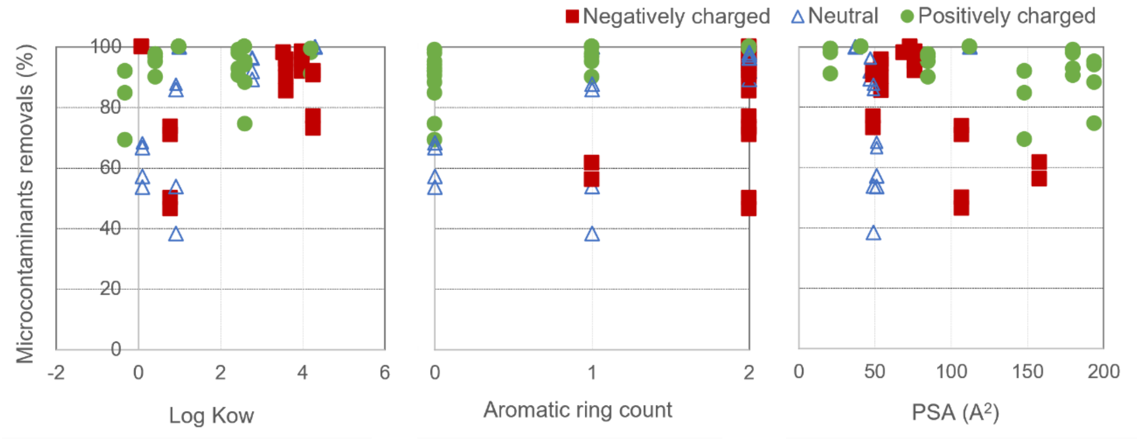

Figure S1. Pharmaceuticals' removal with PAC/(Alum)/MF (spikings 8 and 10) vs. their hydrophobicity (measured by Log Kow) (**left**), aromatic ring count (**middle**) or polar surface area (**right**).
